# PACAP-PAC1 Receptor Activation Is Necessary for the Sympathetic Response to Acute Intermittent Hypoxia

**DOI:** 10.3389/fnins.2019.00881

**Published:** 2019-08-21

**Authors:** Melissa M. J. Farnham, Vikram J. Tallapragada, Edward T. O’Connor, Polina E. Nedoboy, Bowen Dempsey, Suja Mohammed, Angelina Y. Fong, Mandy S. Y. Lung, Fatemeh Derakhshan, Richard J. A. Wilson, Paul M. Pilowsky

**Affiliations:** ^1^The Heart Research Institute, Newtown, NSW, Australia; ^2^Faculty of Medicine, Macquarie University, North Ryde, NSW, Australia; ^3^Department of Physiology, Faculty of Medicine, University of Sydney, Sydney, NSW, Australia; ^4^Department of Physiology and Pharmacology, Hotchkiss Brain Institute and Alberta Children’s Hospital Research Institute, University of Calgary, Calgary, AB, Canada; ^5^Department of Physiology, Faculty of Medicine, Dentistry and Health Sciences, University of Melbourne, Melbourne, VIC, Australia

**Keywords:** Sprague-Dawley rat, mice, anaesthetized, PACAP, sympathetic, intermittent hypoxia, intrathecal

## Abstract

Repetitive hypoxia is a key feature of obstructive sleep apnoea (OSA), a condition characterized by intermittent airways obstruction. Patients with OSA present with persistent increases in sympathetic activity and commonly develop hypertension. The objectives of this study were to determine if the persistent increases in sympathetic nerve activity, known to be induced by acute intermittent hypoxia (AIH), are mediated through activation of the pituitary adenylate cyclase activating polypeptide (PACAP) signaling system. Here, we show that the excitatory neuropeptide PACAP, acting in the spinal cord, is important for generating the sympathetic response seen following AIH. Using PACAP receptor knockout mice, and pharmacological agents in Sprague Dawley rats, we measured blood pressure, heart rate, pH, PaCO_2_, and splanchnic sympathetic nerve activity, under anaesthesia, to demonstrate that the sympathetic response to AIH is mediated via the PAC1 receptor, in a cAMP-dependent manner. We also report that both intermittent microinjection of glutamate into the rostroventrolateral medulla (RVLM) and intermittent infusion of a sub-threshold dose of PACAP into the subarachnoid space can mimic the sympathetic response to AIH. All the sympathetic responses are independent of blood pressure, pH or PaCO_2_ changes. Our results show that in AIH, PACAP signaling in the spinal cord helps drive persistent increases in sympathetic nerve activity. This mechanism may be a precursor to the development of hypertension in conditions of chronic intermittent hypoxia, such as OSA.

## Introduction

Obstructive sleep apnoea (OSA) is characterized by collapse of the upper airway, resulting in frequent intermittent episodes of hypoxemia and hypercapnia. OSA affects approximately 10% of the population and is strongly associated with major cardiovascular diseases, including hypertension, stroke and heart failure; in these disorders, the prevalence of sleep apnoea is close to 50% (Bradley and Floras, 2009). The hypoxemic/hypercapnic events cause large sympathetic surges including increases in heart rate, blood pressure and frequent arousal from sleep, leading to persistently elevated sympathetic nerve activity (SNA), i.e., sympathoexcitation (Leung et al., 2012). While sympathoexcitation may be critical for survival in diseases characterized by high risk of acute crisis such as sudden infant death syndrome (SIDS), marked sympathoexcitation is a key feature of hypertension which, in turn, causes target organ damage, atherosclerosis, renal failure, heart failure and stroke. Common to both OSA and hypertension, elevated SNA is evident before the development of hypertension.

To study the physiological effects of sleep apnoea in animals, investigators use acute intermittent hypoxia (AIH; minutes to hour of exposure), AIH with concurrent hypercapnia, or chronic intermittent hypoxia (CIH; weeks of exposure) (Fletcher, 2001). Despite the temporal pattern and magnitude of hypoxia varying considerably between these models, they all result in persistent sympathoexcitation (Kumar et al., 2006; Dick et al., 2007; Coleman et al., 2010; Iturriaga et al., 2010; Xing and Pilowsky, 2010; Roy et al., 2018), which is important since OSA is a hugely variable condition. The longer the period of sympathoexcitation the more likely it will lead to hypertension (Dick et al., 2007; Xing and Pilowsky, 2010). However, the mechanism of this persistent sympathoexcitation is not understood.

As pituitary adenylate cyclase activating polypeptide (PACAP) is essential for normal cardiorespiratory stress responses including apneas in neonates (Cummings et al., 2004; Ferguson et al., 2013; Barrett et al., 2017), we propose that PACAP plays an important role in intermittent hypoxia induced sympathoexcitation in adults. PACAP is an excitatory neurotransmitter present in all parts of the sympathetic pathway from sensors to efferents, including the carotid body, the rostroventrolateral medulla (RVLM), sympathetic preganglionic neurons (SPN), and the adrenal medulla (Lai et al., 1997; Mazzocchi et al., 2002; Farnham et al., 2008, 2011, 2012; Inglott et al., 2011; Roy et al., 2013). Intrathecal infusion of 1 mM PACAP-38 causes large, long lasting sympathoexcitation without any change in blood pressure (Farnham et al., 2008), similar to that seen after AIH. PACAP acts at 3 receptors, PAC1, VPAC1, and VPAC2, and can differentially regulate blood pressure by activating either PAC1 or VPAC receptors in the spinal cord (Inglott et al., 2012). PACAP is closely associated with catecholaminergic regions and is rate-limiting for the release of catecholamines from the adrenal medulla during prolonged stress (Stroth et al., 2013). Neonatal PACAP knockout mice also have reduced brainstem expression of tyrosine hydroxylase (TH) (Arata et al., 2013), the rate limiting enzyme for catecholamine synthesis. Thus, enhanced PACAP neurotransmission in cardiorespiratory pathways may be the underlying cause of the persistent sympathetic efferent activity seen following periods of sleep-disordered breathing, involving intermittent hypoxia, in sleep apnoea patients (Bradley and Floras, 2009). We therefore hypothesized that much of the persistent increase in sympathoexcitation seen following an episode of intermittent hypoxia, is due to PACAP acting at the PAC1 receptor that exerts long-term plastic effects on post-synaptic sympathetic neurons.

It should be noted, however, that the relative importance of central immune cells such as microglia, in mediating sympathoexcitation is unknown. Microglia in the sympathetic nervous system possess receptors for virtually all neurotransmitters, including PACAP (Kapoor et al., 2016), but ascertaining their function is challenging. In conditions of CIH, it is proposed that microglia may be activated by intermittent hypoxia either directly or indirectly, or by a combination of both mechanisms (Kiernan et al., 2016). Whether microglia directly, or indirectly contribute to intermittent hypoxia-induced sympathoexcitation, remains to be investigated.

Here we report that AIH causes persistent sympathoexcitation and increased expression of phosphorylated TH (serine 40) in PACAP-containing C1 cardiovascular neurons in the RVLM. This sympathoexcitation can be blocked with intrathecal administration of the PACAP antagonist, PACAP(6–38) and does not occur in mice lacking the PAC1 receptor. Using intermittent intrathecal application of PACAP or intermittent glutamate stimulation of the RVLM, we show that PACAP signaling within the spinal cord is an important cause of the long-term facilitation of sympathetic activity. These PACAP effects are mediated by the PACAP preferring receptor, PAC1, in a cAMP-dependent manner.

## Materials and Methods

### Animals

Procedures and protocols were approved by the Animal Care and Ethics Committees of Macquarie University, Sydney Local Area Health District, and the University of Calgary and conducted in accordance with the Australian and Canadian codes of practice for the care and use of animals for scientific purposes. Rats are used as the experiments described involve an integrative approach and no artificial models of these systems currently exist. Mice are used as conventional models for investigating the effects of knocking out genes of interest.

Experiments were conducted on adult male Sprague-Dawley (SD) rats (350–500 g; Animal Resource Centre, Perth, Australia) and adult male and female mice (20–70 g; C57BL/J6 background; University of Calgary, Canada) that were PAC1R^+/+^, PAC1R^+/–^ PAC1R^–/–^, VPAC2R^+/+^ or VPAC2^–/–^. The PAC1 receptor mouse colony (Jamen et al., 2000) was provided by Dr. L. Journot (Hannibal et al., 2001). The VPAC2 receptor mouse colony (Harmar et al., 2002) was provided by Dr. A. Harmar (deceased).

### Surgical Preparation

Sprague Dawley (SD; male; *n* = 104) rats, and mice (male and female; *n* = 6 PAC1^+/+^; 5 PAC1^+/–^; 3 PAC1^–/–^; 8 VPAC2^+/+^; 6 VPAC2^–/–^) were anaesthetized with urethane. For the Fos study *n* = 8 SD rats were anaesthetized with sodium pentobarbitone. Complete details of surgical preparation and data acquisition methods are as described elsewhere (Farnham et al., 2008, 2015).

Briefly, the core temperature of all animals was maintained at 37 ± 0.5°C. The right carotid artery and jugular vein were cannulated for measurement of arterial blood pressure and administration of drugs and fluids, respectively. The trachea was cannulated to permit artificial ventilation. The left greater splanchnic sympathetic nerve was isolated and activity recorded. All animals were bilaterally vagotomized (cervical), ventilated with oxygen-enriched room air and paralyzed (rats – pancuronium bromide 0.8 mg/kg i.v., followed by an infusion of 0.8 mg/kg/hr of pancuronium in 0.9% saline at a rate of 2 ml/h; Astra Zeneca, Australia; mice – rocuronium bromide 0.02 ml/h, i.p., 10 mg/ml; Sandoz, Melbourne). In rats, arterial blood was withdrawn and respiratory blood gas (O_2_ and CO_2_) and pH analysis (electrolyte and blood gas analyzer; IDEXX Laboratories, United States) conducted 10 min before any treatment, and during the recording periods. In some groups, an occipital craniotomy was performed, a needle was inserted into the RVLM with tip location confirmed with a rise in blood pressure > 30 mmHg (Gaede and Pilowsky, 2013) in response to a 50 nl injection of glutamate (100 mM; Sigma). For full details of the *in vivo* mouse preparation see Farnham et al. (2015). All recordings were maintained for 60 min following the final stimulus. Blood gas sampling was not possible in mouse as the volume required proved fatal.

### Single Intrathecal Administration of Drugs

For intrathecal administration of drugs (rats), a catheter was inserted into the intrathecal space and advanced caudally from the cisterna magna to the level of vertebra T5/T6. The drugs in Table 1 were administered in a 10 μl infusion and washed in with 6 μl phosphate buffered saline (PBS). Injections were made over a 30–45 s period, as previously described (Farnham et al., 2008). Responses were recorded for 10 min before performing the AIH protocol.

**TABLE 1:** Drug information for bolus intrathecal administration.

**Drug**	**Action**	**Concentration**	**Dose**	**Company**
Phosphate buffered 0.9% saline (PBS)	Vehicle control	10 mM	100 nmol	Sigma
PACAP-38	Agonist for PAC1, VPAC1 and VPAC2 receptors	300 μM	3 nmol	Auspep Pty. Ltd., Australia
PACAP(6–38)	PAC1 antagonist with some actions at VPAC2	1 mM	10 nmol	Auspep Pty. Ltd., Australia
Rp-Diastereomer of Adenosine 3′,5-Cyclic Monophosphorothioate (Rp-cAMP)	PKA inhibitor	100 mM	1 μmol	Sigma
Brefeldin A (BFA)	EPAC inhibitor	1 mM	10 nmol	Sigma
4-(N-Ethyl-N-phenylamino)-1,2 dimethyl-6-(methylamino)pyrimidium chloride (ZD-7288)	HCN channel blocker	3 mM	30 nmol	Sigma

### Acute Intermittent Hypoxia

In the rat, the AIH protocol consisted of 10, 45 s, episodes of 10% O_2_ in N_2_, each separated by a 5 min recovery period (Xing and Pilowsky, 2010).

In mouse, the AIH protocol consisted of 10 episodes of 25–30 s of removal of O_2_ supplementation (i.e., ventilation with room air alone), separated by 2.5 min recovery periods (Farnham et al., 2015).

### Intermittent, Intrathecal, and RVLM Drug Administration

In rat, the intermittent intrathecal drug infusion protocol consisted of 10 infusions of 10 μl, each conducted over 30–45 s, separated by 5 min. The RVLM microinjections consisted of 10 microinjections of 50 nl, separated by 5 min. The drugs used in this series of experiments are described in Table 2.

**TABLE 2:** Drug information for intermittent administration.

**Drug**	**Concentration**	**Dose**	**Site of delivery**	**Company**
Glutamate	100 mM	5 nmol	RVLM	Sigma
PBS	10 mM	100 nmol 500 pmol	Intrathecal RVLM	Sigma
PACAP-38	10 μM	100 pmol	Intrathecal	Auspep Pty. Ltd., Australia
Vasoactive intestinal polypeptide (VIP)	10 μM	1 nmol	Intrathecal	Auspep Pty. Ltd., Australia
				

### *In vivo* Data Acquisition and Analysis

Data were acquired using a CED 1401 ADC system and Spike 2 acquisition and analysis software (v. 7.12; Cambridge, United Kingdom). Recordings of splanchnic SNA (sSNA) were filtered (10–1000 Hz) and amplified (×2000), then rectified, smoothed (τ 1 s), and normalized by subtracting the residual activity after death. The transformed sSNA was used to calculate changes in sympathetic nerve activity following treatment by obtaining the % change in sSNA from baseline (1 min average of sSNA prior to intermittent stimulation). Mean arterial pressure (MAP), heart rate (HR), and sSNA, were analyzed from 1 min blocs taken 10 and 1 min prior to, and 60 min, after intermittent stimuli. Statistical analysis was conducted with GraphPad Prism software (v 7).

The responses at 60 min after AIH or intermittent drug administrations between strains were compared using a one-way ANOVA with *post hoc t*-tests, and Holm-Šidák correction, or unpaired *t*-tests. Statistical tests are described in section Results.

### Perfusion and Tissue Harvest

Rats that underwent an AIH or intermittent infusion protocol and were to be used for the anatomical Fos, PACAP, TH, and pSer40TH study, were perfused transcardially with ice-cold, RNase-free PBS. Brains that were to be used for qPCR were then extracted, the RVLM excised bilaterally from a 1 mm coronal section under sterile and RNase free conditions. The procedure was carried out in cold conditions to avoid thawing of the samples. The RVLM samples were combined and transferred immediately after excision to 1.5 ml Eppendorf tube, snap-frozen in dry-ice-ethanol slurry and stored immediately at −80°C. Animals used for combined *in situ* hybridization and immunohistochemistry and were additionally perfused with 4% PFA, the brains removed and post-fixed for 24 h in 4% PFA. Following post-fixation, brains were sectioned coronally at 40 μm and cryoprotected until use.

### Combined *in situ* Hybridization (ISH) Fluorescence Immunohistochemistry

The combined protocol for free-floating *in situ* hybridization (PACAP-ISH forward: 5′-GGATCCATTTAGGTGACACTATAGAAGTTACGATCAGGA CGGAAACC-3′; PACAP-ISH reverse: 5′-GAATTCTAATAC GACTCACTATAGGGAGATGC-ACGCTTATGAATTGCTC-3′) and immunohistochemistry was performed as previously described (Li et al., 2005; Farnham et al., 2008). The PACAP probe was used at a final concentration of 100 ng/ml and sections were incubated shaking at 58°C overnight. Digoxigenin-labeled probe was detected using an alkaline phosphatase labeled sheep anti-digoxigenin antibody (1:1000, Roche, Switzerland). TH was detected using a mouse anti-TH (1:2000; Sigma–Aldrich Cat# T1299) primary antibody and a Cy3-conjugated donkey anti-mouse (1:500; Jackson ImmunoResearch Labs Cat# 715-167-003 RRID:AB_2340818) secondary antibody. Fos was detected with a rabbit anti-Fos (1:2000; Thermo Fisher Cat#SCZSC-253) primary antibody and a DyLite488-conjugated donkey anti-rabbit (1:500; Jackson ImmunoResearch Labs Cat# 711-485-152 RRID:AB_2492289) secondary antibody. A colorimetric reaction using nitroblue tetrazolium (NBT) (Roche) and 5-bromo-4-chloro-3-indolyl phosphate (BCIP) salts (Roche) in buffer (0.1 M NaCl, 0.1 M Tris.HCl, pH9.5, 0.1 M MgCl_2_, 0.1% Tween-20, 2 mM levamisole) revealed digoxigenin-labeled neurons as somata containing dark purple precipitants. Immunohistochemistry for pSer40TH was performed as previously described (Nedoboy et al., 2016).

### Quantitative PCR (qPCR) for PACAP and NMDA Receptors

RNA extractions were carried out using Direct-zol Miniprep (Zymoresearch, R2050) kit according to recommendations. Only RNA samples with an optical densiometry 260–280 nm absorption ratio higher than 1.95 were used. Total RNA (50 ng) was reverse transcribed into cDNA in a 40 μl reaction. The resulting reverse transcription products were used in subsequent real-time qPCR experiments for the quantification of mRNA expression of PACAP and NMDA receptors. The reference gene was hypoxanthine guanine phosphoribosyl transferase (HPRT) which is reliably expressed under hypoxic conditions (Yao et al., 2012). Primers for PAC1, vasoactive intestinal peptide receptor types 1 and 2 (VPAC1, and VPAC2, respectively) were used previously (Farnham et al., 2012). All primers were designed using Primer 3 software based on published gene sequences. The sequences and properties of each primer pair are shown in Table 3.

**TABLE 3:** Sequences and properties of gene-specific real-time qPCR primers.

**Gene**	**Genebank accession no.**	**Sequence (5′–3′)**	**Amplicon size (bp)**
HPRT 1	NM_012583	(+) GCTTTCCTTGGTCAAGCAGT (-) TCCAACAAAGTCTGGCCTG	103
PAC1	NM_133511	(+) TCTTGAATGGGGAGGTACAGG (-) TCTTGCTCAGGATGGACAGC	150
VPAC1	NM_012685	(+) CAGCAAGATGTGGGACAACC (-) TGCTGCTCATCCAGACTCG	216
VPAC2	NM_017238	(+) CCGAGGATGAGAGTAAGATCACG (-) AGATGGCTCTCAGCATGAAGG	183
NR1a	NM_017010	(+) ACGGGAGTCCAAGGCAGAGA (-) TCGCTTGCAGAAAGGATGAT	117

Real-time qPCR. Each reaction for real-time qPCR experiments contained 2 μl of reverse transcription product, 400 nM of each primer, and 12.5 μl of the 2× Platinum SYBR Green qPCR Master Mix (Qiagen), made up to 26.5 μl with sterile Milli-Q water. After a 95°C denaturation for 10 min, the reactions were cycled 40 times with a 95°C denaturation step for 30 s and 55°C combined annealing and extension step for 1 min with a single fluorescence measurement. A dissociation curve cycle (95°C -1 min, 55°C -30 s, 95°C -30 s) was conducted after the final cycle to verify that only the specific product was amplified.

All qPCR experiments were set up using the relative standard curve method. Samples from six animals from each group were run in duplicate. Results were obtained, normalized and analyzed as described by us previously (Farnham et al., 2012).

### Imaging and Analysis

Sections of the brainstem extending from the caudal pole of the facial nucleus (Bregma -11.6 mm) to the caudal C1/A1 region were examined under both bright-field and fluorescence conditions (AxioImager Z1, Zeiss, Germany). Cells were counted within the RVLM bilaterally on five sections spaced 200 μm apart, extending from Bregma -11.6 mm caudally to Bregma -12.7 mm by operators blinded to the treatment conditions. The RVLM was defined as a triangular area ventral to the nucleus ambiguus, medial to the spinal trigeminal tract and lateral to the inferior olive or the pyramidal tracts. Once all counts were completed and verified, the conditions were unblinded, and the results were plotted as the mean ± SEM at 200 μm intervals. Counts were made for PACAP^+^, TH-ir, pSer40TH-ir, and Fos-ir neurons as well as all double- and triple- labeling combinations.

Images were captured in grayscale with an Axiocam MR3 digital camera. Pseudocoloring was applied to the fluorescence images. The images were adjusted individually for brightness and contrast with Axiovision 4.5 software to best reflect the appearance of the original images.

## Results

### PACAP Acting at PAC1 Receptors at the Level of the Spinal Cord Is Necessary for the Sympathetic Response to AIH

**FIGURE 1:**
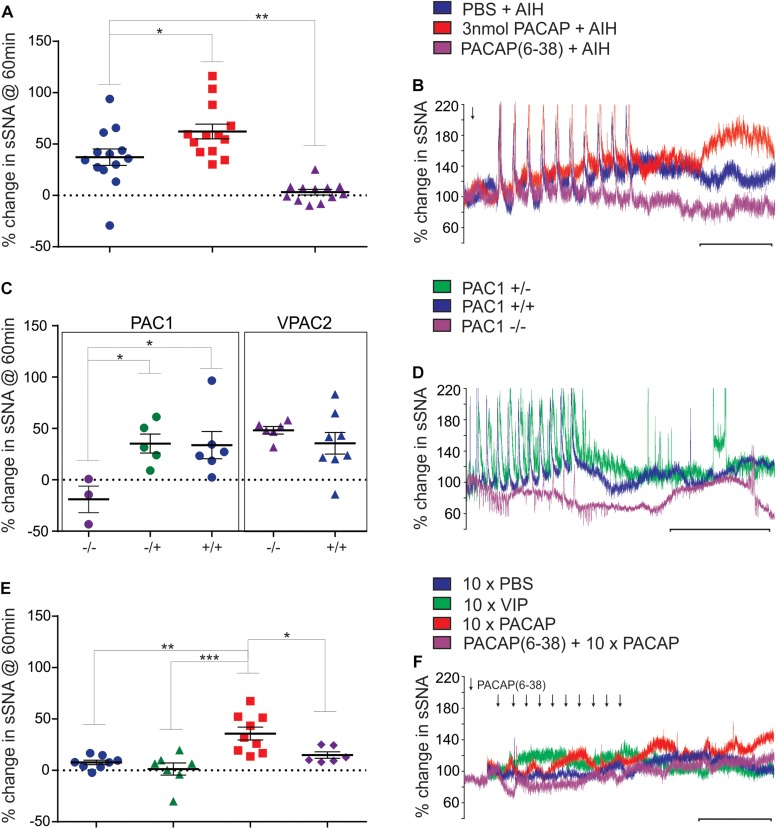
Acute intermittent hypoxia and PAC1 receptor activation causes persistent sympathoexcitation. **(A)** Grouped data (rat) showing the sympathetic response 60 min following AIH treatments after either: intrathecal infusion of PBS (vehicle), 3 nmol PACAP or 10 nmol PACAP(6–38). PACAP (3 nmol) nearly doubles the response to AIH, while the antagonist abolishes the response to AIH. **(B)** Integrated traces of splanchnic nerve recordings (rat) showing the effect of intrathecal infusion of PBS (vehicle), 3 nmol PACAP or 10 nmol PACAP(6–38) and AIH on sympathetic nerve activity. Arrow indicates time of intrathecal infusion. **(C)** Grouped data showing the sympathetic response 60 min following AIH treatment in PAC1 and VPAC2 mouse strains. The PAC1 knockout mouse does not have a sympathetic response to AIH, whereas the PAC1 heterozygous and wild-type strains show a normal response to AIH, as do the VPAC2 strains. **(D)** Splanchnic nerve recordings (mouse) showing the effect of AIH. **(E)** Grouped data (rat) showing the sympathetic response 60 min following intermittent treatments of PBS (vehicle), 1 nmol VIP, 100 pmol PACAP or 10 nmol PACAP(6–38) + intermittent 100 pmol PACAP. Intermittent PACAP, but not VIP, causes a persistent sympathetic response, similar to that seen following AIH. The response to intermittent PACAP is significantly reduced by prior administration of the PACAP antagonist. **(F)** Integrated traces of splanchnic nerve recordings (rat) showing the effect of intermittent intrathecal infusion of PBS (vehicle), 1 nmol VIP, 100 pmol PACAP or 10 nmol PACAP(6–38) + intermittent 100 pmol PACAP on sympathetic nerve activity. In **(B,D,F)** the scale bar represents 30 min and arrows indicate time of intrathecal infusion. ^∗^*P* < 0.05, ^∗∗^*P* < 0.01, ^∗∗∗^*P* < 0.001.

To determine the physiological relevance of PACAP signaling during AIH, a sub-threshold dose (3 nmol) of PACAP (Farnham et al., 2011) 10 min prior to AIH was administered intrathecally. PACAP pre-treatment nearly doubled the sympathetic response 60 min after the 10th hypoxic challenge (*n* = 13; Δ62.3 ± 7.2%; ANOVA with Holm- Šidák correction *P* = 0.02; Figures 1A,B), compared to AIH alone (Δ37.2 ± 8.0%). PACAP was necessary for the AIH-induced sympathoexcitation, since the PACAP antagonist, PACAP(6–38) (10 nmol) completely abrogated this response (Δ3.2 ± 2.7%; ANOVA with Holm- Šidák correction *P* = 0.003; Figures 1A,B). PACAP(6–38) acts primarily at PAC1, does not act at VPAC1, but can have some antagonist effects at VPAC2 receptors (Dickinson et al., 1997).

To further clarify which receptor was responsible for mediating this response, the AIH protocol was conducted in PAC1 and VPAC2 receptor knockout mice and their wildtype littermate controls (Farnham et al., 2015). Strikingly, AIH did not cause an elevation of sympathetic activity in PAC1^–/–^ mice (*n* = 3; Δ-19.0 ± 12.9%; ANOVA with Holm-Šidák correction *P* = 0.047 compared to wild-type; Figures 1C,D). A limitation should be noted here that due to the difficulty in breeding PAC1^–/–^ mice only 3 mice were available for this study. However, the sympathetic response to AIH of heterozygous PAC1^+/–^ mice (*n* = 5; Δ35.3 ± 9.3%; Figures 1C,D) was indistinguishable from that of the wildtype mice (PAC1^+/+^: *n* = 6; Δ33.8 ± 13.3%; ANOVA with Holm- Šidák correction *P* = 0.93), and the rat model described in Figures 1A,B. In contrast, deletion of the VPAC2 receptor (VPAC2 ^–/–^) did not affect AIH-induced sympathoexcitation (*n* = 6; Δ48.3 ± 3.7%; Figure 1C), which was comparable to the wildtype (VPAC2^+/+^: *n* = 8; Δ35.6 ± 10.6%; ANOVA with Holm- Šidák correction *P* = 0.63; Figure 1C). Taken together, these rat and mouse results suggest that the sympathetic long term facilitation (LTF) response to AIH is caused by activation of the PAC1 receptor.

### Intermittent PACAP Acting at PAC1 Receptors in the Spinal Cord, Is Sufficient to Cause LTF in the Absence of AIH

Next, we determined if intermittent PACAP can elicit a sympathetic response qualitatively similar to that seen following AIH. First, determined if PACAP was sufficient to cause sympathoexcitation by administering 10 intermittent doses (100 pmol) of PACAP (to a total dose of 1 nmol that was well below threshold for a sympathetic response when delivered as a bolus (Farnham et al., 2011). Each 10 μl infusion was delivered at a 5 min interval (*n* = 9). This protocol evoked sympathoexcitation (Δ35.8 ± 6.2%; Figures 1E,F) to a level that was similar to that observed following AIH. The results demonstrate that intermittent administration of PACAP alone, at a sub-threshold dose at the level of the spinal cord, is sufficient to cause marked sympathoexcitation. Importantly, pre-treatment with the PACAP antagonist [10 nmol PACAP(6–38)] prior to intermittent PACAP significantly reduced (*n* = 6; Δ14.9 ± 3.2%; ANOVA with Holm- Šidák correction *P* = 0.04 Figures 1E,F) the sympathoexcitatory response to intermittent PACAP. On the other hand, intermittent application of VIP (1 nmol; a VPAC1/2 agonist) did not affect sympathetic activity (*n* = 7; Δ1.3 ± 5.9%; Figures 1E,F) and was equivalent to intermittent infusion of PBS (vehicle control; *n* = 8; Δ7.7 ± 2.2%; ANOVA with Holm- Šidák correction *P* = 0.90; Figures 1E,F). These data demonstrate that PACAP, acting at the PAC1 receptor, mediates the sympathoexcitatory response.

### Fos, PACAP and NMDA1a Receptor Expression, in Rat RVLM, Is Unaltered by AIH, but pSer40TH Expression Is Elevated

**FIGURE 2:**
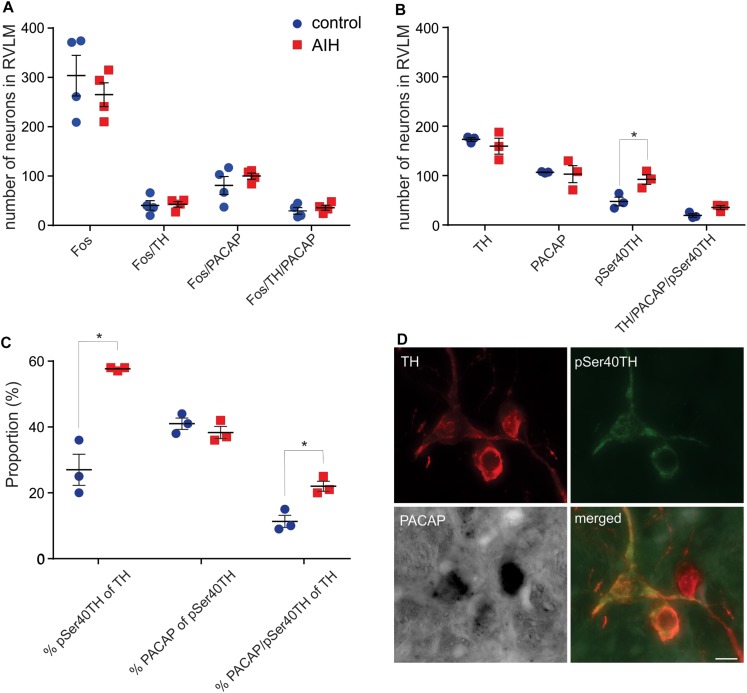
C1 neurons in rat RVLM are activated by AIH. **(A)** Mean ± SEM number of neurons in RVLM stained for Fos, TH and PACAP mRNA 60 min after control (*n* = 4) or AIH (*n* = 4). There are no differences in Fos staining, in any population, between the two treatment groups. **(B)** Neurons in RVLM (mean ± SEM) stained for pSer40TH, TH and PACAP mRNA 60 min after control (*n* = 3) or AIH (*n* = 3). Number of neurons expressing pSer40TH doubled in the AIH treated group compared to control. **(C)** The data from **(B)** is expressed as proportion of TH or pSer40TH populations. AIH caused a doubling in the proportion of TH neurons that also expressed pSer40TH (27 ± 5% vs. 58 ± 0.3%) and a doubling in the proportion of TH neurons that also contained pSer40TH and PACAP mRNA. **(D)** Micrographs with 3 neurons, from an AIH treated rat, stained for TH, pSer40TH and PACAP mRNA, and a merged image. All 3 TH neurons are positive for PACAP mRNA but only 2 are pSer40TH positive. RVLM – rostral ventrolateral medulla. Scale bar represents 10 μm. ^∗^*P* < 0.05.

Previously we showed that the 80% of spinally projecting, TH neurons contain PACAP mRNA (Farnham et al., 2008) suggesting that the source of the AIH-induced PACAP in the spinal cord is from the presympathetic RVLM neurons. To determine which neurons in the RVLM are activated during AIH, we subjected sodium pentobarbitone anaesthetized rats, to the AIH protocol (*n* = 4). Sodium pentobarbitone was used because urethane is well known to cause substantial Fos expression in the absence of any challenge. Control rats (*n* = 4) were prepared in the same way but did not undergo the AIH protocol. After the experiment, rats were perfused, and the brains removed. Sections were processed for PACAP mRNA *in situ* hybridization, TH-immunoreactivity (ir) and Fos-ir. Somewhat surprisingly, AIH did not cause an increase in the numbers of neurons expressing Fos in the RVLM (304 ± 41 vs. 265 ± 24; Unpaired *t*-test *P* = 0.45) but this was also reported by another group (Herr et al., 2013). There was also no change in Fos expression within the TH population (41 ± 10 vs. 43 ± 6; Unpaired *t*-test *P* = 085; Figure 2A) or the PACAP population (81 ± 18 vs. 100 ± 6; Unpaired *t*-test *P* = 0.36). There was also no change in PACAP receptor mRNA or NMDA1a receptor mRNA collected from RVLM punches of rats that had undergone AIH (*n* = 6), intrathecal PACAP prior to AIH (*n* = 6), intrathecal PACAP antagonist prior to AIH (*n* = 6), intrathecal intermittent PACAP (*n* = 6) and intrathecal intermittent PBS (*n* = 6); quantitative real-time PCR (qPCR), data not shown.

To approach the question of neuronal activation from a different angle we used an antibody that we developed against TH that was phosphorylated at Serine 40 and showed a spatial expression pattern, different to TH, after 2 h of hypotension (Nedoboy et al., 2016), suggesting that it may be an alternative marker of activation of catecholaminergic neurons. Since expression of phosphorylated TH is unaffected by urethane anaesthesia, and to match the anatomy with the experimental condition used for the physiological results, we performed the AIH protocol (*n* = 3) and compared it with control (*n* = 3) on another set of urethane-anaesthetized rats for immunoreactivity to TH and pSer40TH (Figure 2C). In the control state we found that 28% of TH neurons in the RVLM were phosphorylated (serine40) as described previously (Nedoboy et al., 2016). AIH had no effect on the overall number of TH or PACAP positive neurons but increased the number of phosphorylated TH (pSer40TH) neurons (of which 41% are PACAP expressing) by 93% (48 ± 9 vs. 92 ± 10 neurons; Unpaired *t*-test *P* = 0.005; Figure 2B). When focused on the TH and pSer40TH populations we found that 27% of TH neurons expressed pSer40TH in the control condition and this doubled to 58% following AIH (unpaired *t*-test *P* < 0.0001; Figure 2C). Likewise, 11% of TH neurons contained both pSer40TH and PACAP mRNA under control conditions, which doubled to 22% following AIH (Unpaired *t*-test *P* = 0.009; Figures 2C,D) indicating that the PACAP neurons in the RVLM may be being activated.

### Intermittent Activation of RVLM Neurons Is Sufficient to Cause Sympathoexcitation in the Absence of AIH and Is Dependent on PACAP Release in the Spinal Cord

**FIGURE 3:**
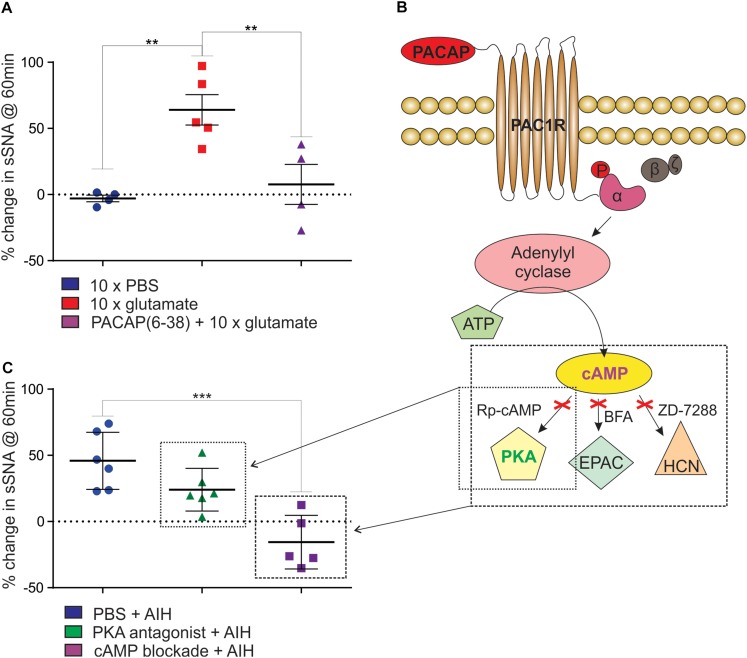
Intermittent activation of rat RVLM mimics AIH, mediated by PACAP in a cAMP-dependent manner. **(A)** Intermittent injections of 5 nmol glutamate in the RVLM cause a persistent increase in sympathetic nerve activity, which does not occur after intermittent injection of the vehicle control, PBS. The sympathetic response following intermittent glutamate injections is abolished by prior intrathecal infusion of the PACAP antagonist, 10 nmol PACAP(6–38). **(B)** An illustration of the intracellular signaling pathway used by PACAP. When PACAP binds to the PAC1 G-protein coupled receptor, the α-subunit of the G-protein is phosphorylated (“P”), adenylyl cyclase is then activated causing the conversion of ATP into cAMP. cAMP acts at 3 downstream effector proteins, Protein Kinase A (PKA), Exchange Protein Activated by cAMP (EPAC) and hyperpolarization-activated cyclic nucleotide-gated (HCN) channels. The activity of cAMP can be blocked by using a cocktail of 1μmol Rp-Diastereomer of Adenosine 3′,5-Cyclic Monophosphorothioate (Rp-cAMP), 10 nmol Brefeldin A (BFA) and 30 nmol 4-(N-Ethyl-N-phenylamino)-1,2 dimethyl-6-(methylamino)pyrimidium chloride (ZD-7288) to block the three downstream effector proteins. **(C)** When PKA alone is blocked using 1 μmol Rp-cAMP (small box in **B**) there is no significant reduction in the response to AIH. When cAMP is blocked with 1 μmol Rp-cAMP, 10 nmol BFA and 30 nmol ZD-7288 (large box in **B**), the sympathetic response to AIH is abolished. ^∗∗^*P* < 0.01, ^∗∗∗^*P* < 0.001.

We next determined if neurons in the RVLM, including C1 neurons that project to the spinal cord, are responsible for the PACAP-mediated effects described above. Since TH in C1 neurons was phosphorylated in response to AIH, demonstrating an increase in activation, we aimed to determine if the sympathetic response to AIH was due specifically to the effects of hypoxia, or more generally due to intermittent stimulation of sympathoexcitatory pathways. We found that intermittent microinjection of glutamate (5 nmol) into the RVLM caused persistent sympathoexcitation (*n* = 5; Δ64.1 ± 11.5%; ANOVA with Holm- Šidák correction *P* = 0.005; Figure 3A), compared to intermittent microinjection of PBS (*n* = 4; Δ-3.0 ± 2.5%; Figure 3A) as described previously (Kakall et al., 2018). This sympathetic response was qualitatively similar to that induced by AIH. Significantly, the RVLM-glutamate induced sympathoexcitation was abolished by prior intrathecal infusion of the PACAP antagonist (10 nmol; *n* = 4; Δ7.7 ± 15.1%; ANOVA with Holm- Šidák correction *P* = 0.01; Figure 3A). In summary, the findings suggest that the persistent sympathoexcitation induced by AIH is due to intermittent activation of PACAP-containing bulbospinal sympathoexcitatory cardiovascular pathways and does not require direct effects of hypoxia on sympathetic neurons.

### Sympathoexcitation Following AIH Is Mediated by cAMP

Finally, we determined if the sympathoexcitation following AIH is mediated through cAMP signaling pathway as the PAC1 receptor is primarily coupled to cAMP via Gαs (Vaudry et al., 2000). As there is no single pharmacological blocker of cAMP we intrathecally administered a combination of antagonists to the downstream effector proteins of cAMP – PKA, EPAC, and HCN channels at previously described concentrations (Tallapragada et al., 2016). Administration of the antagonists had no effect on resting sympathetic tone prior to AIH and had no effect on the responses to each hypoxic bout. We found that blocking the cAMP pathway (1 μmol Rp-cAMP + 10 nmol BFA + 30 nmol ZD-7288; Table 1 and Figure 3B) abolished the sympathetic response to AIH (*n* = 5; Δ-15.6 ± 9.0%; ANOVA with Holm- Šidák correction *P* = 0.0003; Figure 3C) compared to AIH alone. As PKA is the primary effector of cAMP we also administered the PKA blocker (1 μmol Rp-cAMP) on its own prior to AIH (*n* = 6), but did not find any significant change in the sympathetic response to AIH (Δ24.0 ± 6.6%; ANOVA with Holm- Šidák correction *P* = 0.14; Figure 3C), suggesting that although the response requires cAMP, it is not solely dependent on the PKA pathway.

### All Increases in Sympathetic Activity Following Intermittent Stimulation of Cardiovascular Areas Occur in the Absence of Any Changes in Blood Pressure, pH or PaCO_2_

A short duration of intermittent hypoxia (i.e., AIH) is known to cause increased sympathetic activity without any change in mean arterial pressure (Dick et al., 2007; Xing and Pilowsky, 2010) a result confirmed here (Table 4). Changes in pH and PaCO_2_ can also affect sympathetic nerve activity and confound the results. Blood gases were monitored throughout the experiments (except for the mice, as the blood volume is too small) to ensure that the increases in sympathetic nerve activity observed following AIH were not due to the stimulatory effects of CO_2_. Table 4 shows that PaCO_2_ and pH were within the physiological range at the end of the recording period for all treatment groups.

**TABLE 4:** MAP and metabolic parameters were unchanged 60 min after intermittent stimulation.

**Treatment group**	**N (animals)**	**Baseline MAP (mean ± SEM)**	**Change in MAP (mean ± SEM) @ 60 min**	**pH @ 60 min**	**PaCO_2_ @ 60 min**
**RAT SPINAL CORD STUDIES**					
PBS + AIH	13	88 ± 5	6 ± 2	7.42 ± 0.01	39 ± 2
3 nmol PACAP + AIH	13	95 ± 4	3 ± 3	7.42 ± 0.01	40 ± 1
PACAP(6–38) + AIH	12	97 ± 4	−5 ± 5	7.41 ± 0.01	40 ± 1
10 × PBS	8	95 ± 3	−2 ± 3	7.41 ± 0.01	40 ± 2
10 × PACAP	9	95 ± 6	0 ± 4	7.40 ± 0.01	40 ± 2
10 × VIP	7	98 ± 3	−4 ± 4	7.41 ± 0.01	41 ± 2
PACAP(6–38) + 10 × PACAP	6	104 ± 2	−5 ± 3	7.45 ± 0.03	37 ± 2
**RAT RVLM STUDY**					
10 × PBS	4	118 ± 8	−18 ± 6	7.41 ± 0.01	43 ± 1
10 × glutamate	4	101 ± 4	−3 ± 3	7.44 ± 0.002	41 ± 1
PACAP(6–38)+10 × glutamate	4	106 ± 9	−11 ± 5	7.44 ± 0.01	42 ± 2
**RAT CAMP STUDY**					
PBS + AIH	6	101 ± 4	0 ± 8	7.41 ± 0.01	42 ± 1
cAMP block + AIH	5	103 ± 6	0 ± 5	7.45 ± 0.01	38 ± 2
PKA block + AIH	6	110 ± 8	2 ± 5	7.42 ± 0.04	40 ± 1
**MOUSE STUDY**					
PAC1 +/+	5	58 ± 5	−2 ± 3	–	–
PAC1 ©	5	61 ± 5	−11 ± 4	–	–
PAC1 -/-	3	45 ± 5	−5 ± 3	–	–
VPAC2 +/+	7	70 ± 6	−15 ± 4	–	–
VPAC2 -/-	5	57 ± 2	−7 ± 4	–	–

## Discussion

We report here for the first time that the sympathoexcitation caused by AIH is likely mediated, at least in part, by central neuroplasticity involving PACAP acting on PAC1 receptors in the spinal cord. Our results support that of Xing and Pilowsky (2010), with the increase in SNA occurring without any significant change in MAP, PaCO_2_, or pH. Mimicking the response seen after AIH, sub-threshold intermittent intrathecal PACAP alone is sufficient to generate a prolonged sympathetic response, as is intermittent activation of the RVLM with glutamate (Kakall et al., 2018). Using pharmacological agents and PACAP receptor knockout mice, we demonstrate that the sympathetic response to AIH is mediated via spinal PAC1 receptors and dependent on cAMP.

Sustained hypertension is a serious disorder with devastating individual and community consequences that include: vascular disease, heart failure and stroke. Hypertension is usually associated with elevated sympathetic nerve activity (SNA) and increased activity of presympathetic neurons in the RVLM (Chan et al., 1991; Minson et al., 1996). An outstanding question in this field is, how do sympathetic pathways and their neurotransmitter systems cause this persistent change? One hypothesis lies in the pattern of neurotransmitter signaling.

Intermittent activation of neuronal pathways is more potent in enhancing neuronal activity and having long term consequences than continuous activation (Fuller et al., 2003; Kumar et al., 2006; Peng et al., 2009; Roy et al., 2018); phenomena that are the basis for memory formation and are well-known at the behavioral (gambling) and cellular (long-term potentiation) levels, but relatively uncharacterized in cardiovascular control. Yet, repetitive, intermittent hypoxic events are the hallmark of OSA which is characterized by persistent sympathoexcitation and now regarded as the leading cause of secondary hypertension (Pedrosa et al., 2011).

While the pathophysiology of sympathoexcitation and hypertension in OSA are not completely understood, current evidence suggests there are multiple sites contributing to the persistent effects of intermittent hypoxia. For example, intermittent hypoxia with concurrent hypercapnia acts at the carotid body to cause robust increases in afferent activity to the brain which is also reflected in the long-lasting sympathetic output (Roy et al., 2018). This activity is dependent on TRPV1 and P2X receptors. Other long term effects of intermittent hypoxia appear to involve reactive oxygen species (Prabhakar et al., 2012), angiotensin (Kim et al., 2018), and serotonin (Ling et al., 2001) to name a few. Intermittent stimulation of the RVLM also causes a persistent sympathoexcitation (Kakall et al., 2018), but what has remained elusive is the identity of the final central output driving the increase in sympathetic activity.

PACAP is found almost unchanged in every vertebrate species examined (Hannibal, 2002; Vaudry et al., 2009) and is generally excitatory. PACAP binds to three G-protein coupled receptors, PAC1, VPAC1, and VPAC2 (all of which have many splice variants). The PACAP receptors are primarily coupled to either Gαs or Gαq and activate the cAMP pathway to exert short- and long- term changes in neuronal activity and gene expression (Farnham and Pilowsky, 2010; Harmar et al., 2012). The presence of PACAP and its receptors throughout the cardiorespiratory sympathetic circuit, from the carotid body (Roy et al., 2013) and RVLM, to sympathetic post-ganglionic neurons and the adrenal medulla in the periphery, indicates that this peptide plays an important role in blood pressure regulation (Farnham and Pilowsky, 2010). Consistent with a role in blood pressure regulation, activation of PACAP receptors regulate catecholamine gene expression (Hong et al., 1998; Choi et al., 1999; Park et al., 1999; Borba et al., 2005), TH activity which is rate limiting for noradrenaline (and adrenaline) synthesis (Tonshoff et al., 1997; Hamelink et al., 2002; Bobrovskaya et al., 2007), and release of acetylcholine at neuronal nicotinic synapses (Pugh et al., 2010). Our finding that AIH increased the number of phosphorylated TH neurons in the RVLM is consistent with this literature and may explain why antagonism of RVLM PACAP receptors blocked the intermittent glutamate-induced sympathoexcitation (Kakall et al., 2018). Interestingly, while AIH increased the number of phosphorylated TH neurons in the RVLM within 60 min, the number of Fos positive neurons over the same time window was unchanged. This indicates that pSer40TH may be a more precocious, sensitive and/or persistent marker of neuronal activation in the C1 population than Fos.

Notwithstanding a role for PACAP signaling in the RVLM, the results herein suggest that the sympathoexcitation observed following AIH is due, at least in part, to PACAP triggering additional plastic changes via spinal PAC1 receptors. Specifically, our data demonstrate that persistent sympathoexcitation can be induced by AIH *and* intermittent stimulation of the RVLM in the absence of hypoxic events; and in both cases, sympathoexcitation is dependent upon activation of PAC1 receptors in the spinal cord. Moreover, the similarity of the time-course of action of PACAP on SNA when administered intermittently into the spinal cord with the time-course of the response to AIH, and the finding that blocking spinal PACAP receptors abolishes this response, highlights the likely importance of spinal PACAP signaling in this physiological condition. Accordingly, PACAP-mediated plasticity in the spinal cord might further exacerbate and prolong neuronal excitability caused by plasticity in the carotid body and RVLM, giving rise to long term sympathoexcitation which, in turn, may contribute significantly to the hypertension that accompanies OSA.

Indeed, PACAP may be an important mediator of the hypertensive response observed in OSA. Whereas AIH causes sympathoexcitation without any change in blood pressure, experimental CIH – a well-established model for sleep apnoea – causes hypertension which is accompanied by increased noradrenaline release from the adrenal medulla (Kumar et al., 2006). In this respect, we note that a high dose of intrathecal PACAP can elevate blood pressure directly by activating PAC1 receptors on SPN innervating noradrenaline-secreting adrenal medulla chromaffin cells (Inglott et al., 2012) and that PACAP knockout mice, a proposed model of Sudden Infant Death Syndrome (SIDS) with blunted cardiorespiratory responses to hypoxia (Cummings et al., 2004), also have reduced TH expression (Arata et al., 2013).

### Neurons or Glia?

PACAP receptors are present on blood vessels, microglia, astrocytes, and neurons, and therefore the question arises, which of these cell types mediates the reported effects? The cerebral and pial vasculature primarily expresses VPAC1 receptors (Fahrenkrug et al., 2000) which are unaffected by PACAP(6–38), and are therefore unlikely to contribute to the PACAP-mediated sympathetic responses to AIH. Microglia are also unlikely to contribute for several reasons: (1) Activation of microglial VPAC1 and PAC1 receptors *reduce* the severe cardiovascular consequences of acute kainic acid-induced seizure (Delgado et al., 2002; Bhandare et al., 2015; Bhandare et al., 2016). This contrasts with PACAP-mediated *prolongation* of the milder cardiovascular consequences of AIH. (2) Only a fraction of microglia (1–20%) respond to neuropeptides, even when in an activated state (Pannell et al., 2014). (3) Even in chronic conditions of intermittent hypoxia the activation state and function of microglia is unclear (Kiernan et al., 2016). Therefore, the effects we see in our AIH model following activation or blockade of PAC1 receptors are unlikely due to activation of microglia.

Astrocytes may have a role in PACAP-mediated sympathetic responses to AIH because they are important for maintaining long lasting PACAP-induced dorsal horn-mediated neuropathic pain (Yokai et al., 2016). However, only a few astrocytes express PAC1 receptors (Yokai et al., 2016). Thus, while astrocytes may have an indirect role, PACAP receptors on neurons remain the most likely mediators of long-term sympathetic responses to AIH.

### Technical Considerations

Our electrophysiological data are drawn from an anesthetized and vagotomized preparation used widely to study sympathetic activity. Indeed, there were good reasons for each of these conditions. Urethane was used to ensure the preparation stability required to maintain long term (>2 h) splanchnic nerve recordings; vagotomy prevented sensory information from lung stretch receptors caused by mechanical ventilation; and we cut the splanchnic nerve distal to the recording electrode to ensure only efferent (sympathetic) activity was monitored. Nonetheless, each of these maneuvers may have caused unforeseen effects on sympathetic activity, such as accentuating the short or long-term effects of hypoxia. The effects of the surgical preparation may also have affected Fos expression, thereby masking any AIH-induced changes.

Another important caveat is that infusion of solutions into the intrathecal space creates the risk of diffusion of the solution back up the spinal cord, to the RVLM. To minimize this risk, we used small injection volumes, positioned the animals with their heads elevated in relation to the thoracic spinal cord and, in previous studies, used dye and spinal cord transections to confirm that diffusion to the brainstem does not occur (Inglott et al., 2011). In addition, as PACAP is rapidly broken down by dipeptidyl peptidase IV (DPP-IV) with a half-life ∼ 2 min (Zhu et al., 2003) and DPP-IV is expressed in the CSF (Kato et al., 1979), we judge the possibility of intrathecal PACAP diffusing to, and activating, the RVLM as being highly unlikely.

## Conclusion

The PACAP signaling system may be a useful therapeutic target, not only for sleep apnoea sufferers, but in other conditions of repetitive insults, as persistent sympathoexcitation was generated in the absence of intermittent hypoxia. Little is known about neuroplasticity in the cardiovascular system and further information is vital if we are to understand disorders such as sleep apnoea, that cause hypertension leading to target organ damage, heart failure, vascular disease, renal failure and stroke. A better understanding of how different neurotransmitters operate in central cardiorespiratory pathways will lead to precision-targeted and effective treatments for many autonomic disorders including sleep-apnoea-induced hypertension.

## Data Availability

The raw data supporting the conclusions of this manuscript will be made available by the authors, without undue reservation, to any qualified researcher.

## Ethics Statement

Animal Subjects: The animal study was reviewed and approved by the Sydney Local Area Health District Animal Care and Ethics Committee, Macquarie University Animal Care and Ethics Committee, and University of Calgary Animal Care and Ethics Committee.

## Author Contributions

MF, PP, and RW designed the experiments, interpreted the results, and prepared the manuscript. MF, AF, and ML performed the Fos study. BD acquired all the microscopic images for the Fos study and performed the initial count, which was verified by VT and analyzed by MF. MF and VT performed and analyzed the AIH and intermittent drug experiments in rat. SM performed, analyzed, and interpreted the quantitative real-time PCR experiments, and designed and constructed the PACAP riboprobes for *in situ* hybridization. MF and EO’C performed and analyzed the AIH experiments in mice. MF and PN performed and analyzed the TH/pSer40TH data and, with FD, the intermittent glutamate data. MF and PN prepared the figures. All authors critically reviewed and commented on the manuscript.

## Conflict of Interest Statement

The authors declare that the research was conducted in the absence of any commercial or financial relationships that could be construed as a potential conflict of interest.
